# Supplementary material for: A metabologenomics approach reveals the unexplored biosynthetic potential of bacteria isolated from an Amazon Conservation Unit
Source: Microbiol Spectr. 2024 Dec 10;13(1):e00996-24. doi: 10.1128/spectrum.00996-24 (PMC11705897; doi:10.1128/spectrum.00996-24)
Supplement: Supplemental material — Fig. S1 to Fig. S3: Table S1. [file spectrum.00996-24-s0001.docx]

Supplementary figure 1: Utinga State Park (dark green). Sampling points are identified as A, B and C.


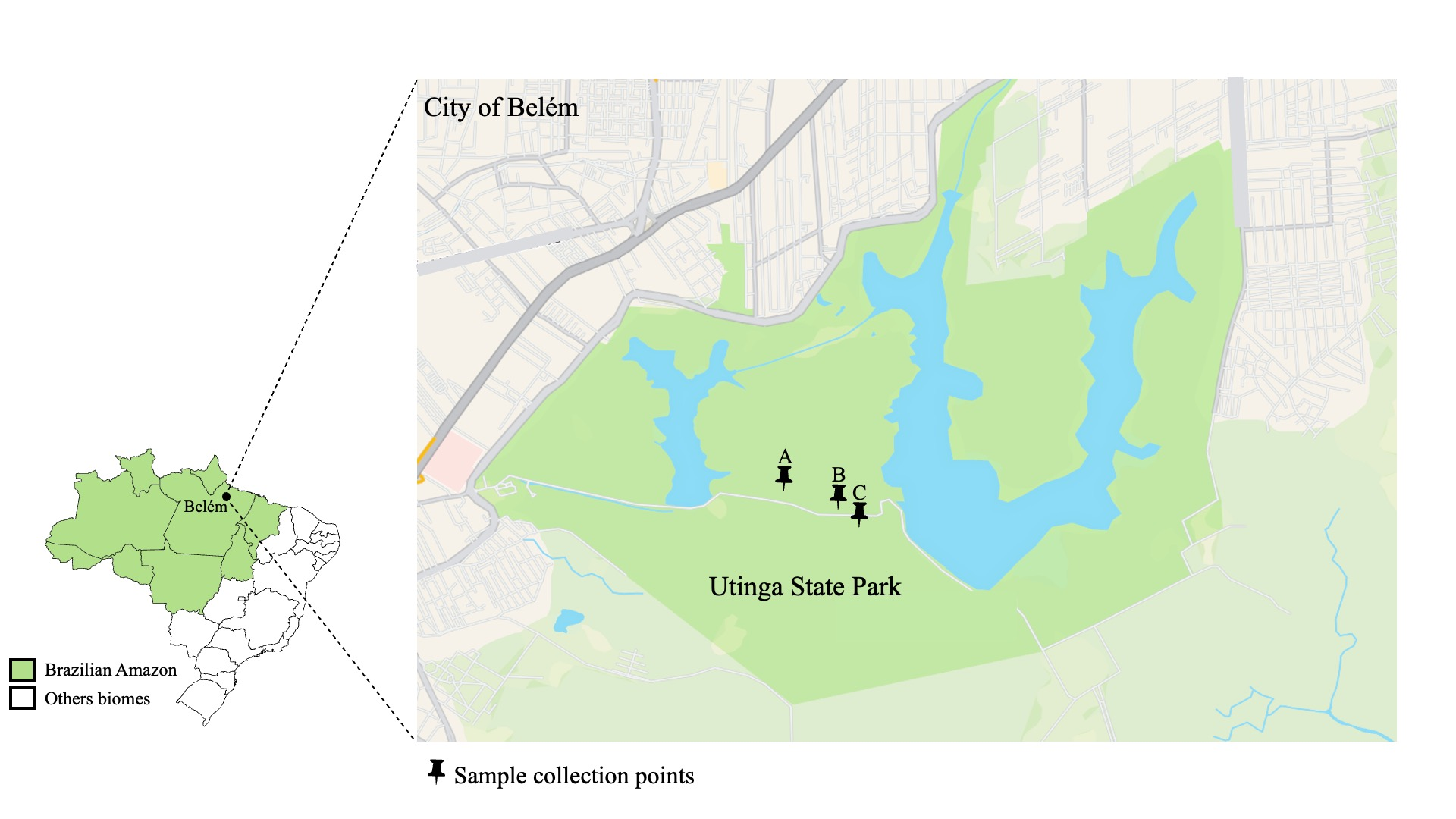


**Supplementary figure 2**: Circular map of *Rhodococcus* sp. ACT016 genome (contig 1 and contig 2). From the outermost ring to the innermost: locations of the 19 Biosynthetic Gene Clusters; genome size; Coding Sequences (forward and reverse); the genomes of Rhodococcus sp. W8901; Prescottella equi ATCC 3; ; *P. equi* BJ13; *P. equi* DSSKP-R-001; *P. equi* FDAARGOS 952; *P. equi* JCM94-14; *P. equi* JCM94-27; *P. equi* JCM94-31; *P. equi* JCM94-3; *P. equi* JID03-46; *P. equi* JID03-56; *P. equi* PAM2287; and *P. equi* U19; G+C skew and G+C content.


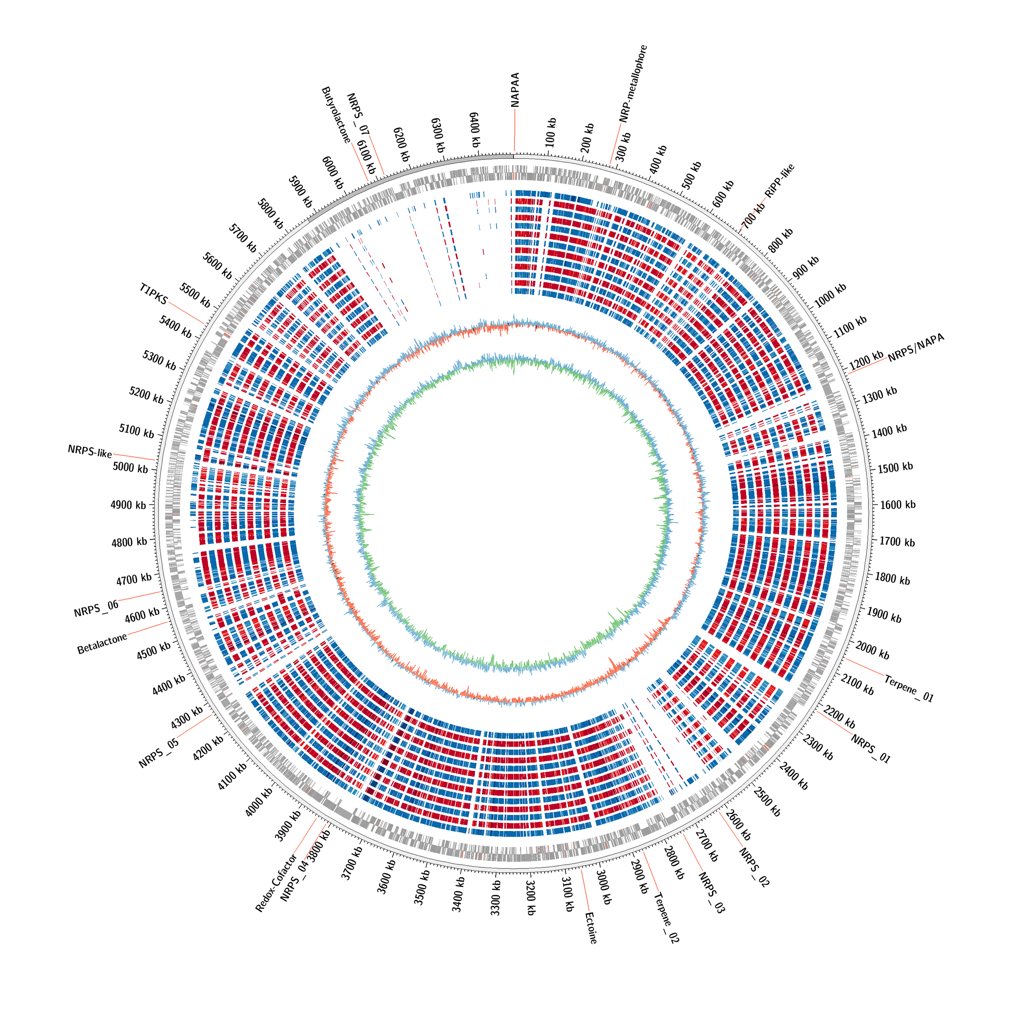


Supplementary figure 3: Analysis carried out on contig 2 of the Rhodococcus sp. ACT016 genome.


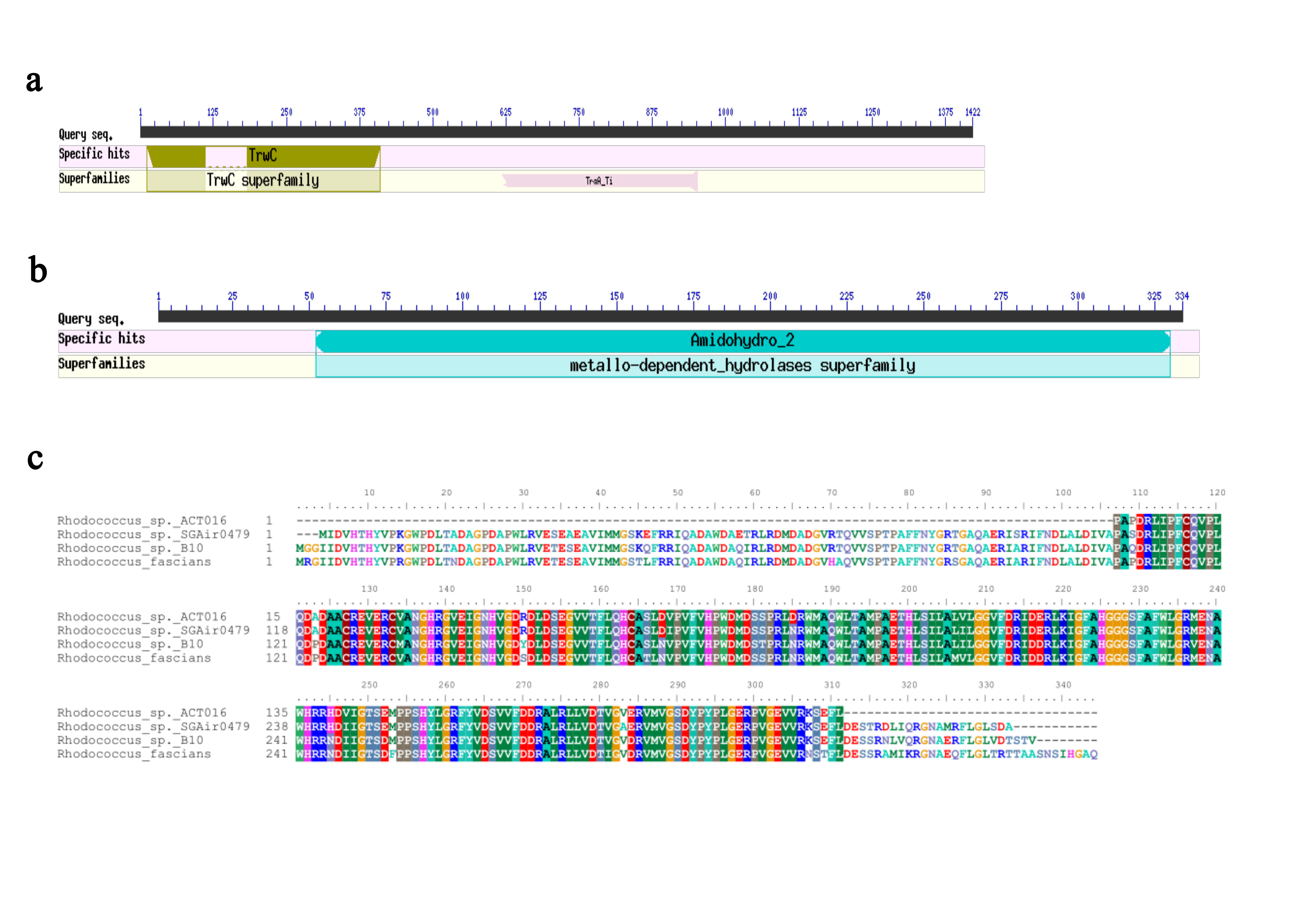


Supplementary table 1: References used in comparative genomics analysis for the three strains.

| Reference genome | Access Number | Identity (%)* |
| --- | --- | --- |
| *Streptomyces* cadmiisoli ZFG47 | CP030073.1 | 99.15 |
| *Streptomyces* griseoviridis F1-27 | CP034687.1 | 99.87 |
| *Streptomyces* griseoviridis K61 | CP029078.1 | 99.87 |
| *Streptomyces* koyangensis SCSIO 5802 | CP049945.1 | 99.21 |
| *Streptomyces* koyangensis VK-A60T | CP031742.1 | 99.28 |
| *Streptomyces* sp. HF10 | CP047144.1 | 99.21 |
| *Streptomyces* sp. NEAU-sy36 | CP058977.1 | 99.28 |
| *Streptomyces* sp. SUK 48 | CP045740.1 | 99.02 |
| *Streptomyces* sp. WA1-19 | CP085039.1 | 99.08 |
| *Streptomyces* sp. WP-1 | CP123923.1 | 99.21 |
| *Rhodococcus* sp. W8901 | CP054690.1 | 99.14 |
| *Prescottella* equi ATCC 33701 | AP025268.1 | 98.68 |
| *Prescottella* equi BJ13 | CP118697.1 | 98.68 |
| *Prescottella* equi DSSKP-R-001 | CP027793.1 | 98.68 |
| *Prescottella* equi FDAARGOS 952 | CP065594.1 | 98.68 |
| *Prescottella* equi JCM94-14 | AP024181.1 | 98.68 |
| *Prescottella* equi JCM94-27 | AP024187.1 | 98.68 |
| *Prescottella* equi JCM94-31 | AP024189.1 | 98.68 |
| *Prescottella* equi JCM94-3 | AP024192.1 | 98.68 |
| *Prescottella* equi JID03-46 | AP024196.1 | 98.68 |
| *Prescottella* equi JID03-56 | AP024198.1 | 98.68 |
| *Prescottella* equi PAM2287 | CP095477.1 | 98.68 |
| *Prescottella* equi U19 | AP025544.1 | 98.68 |
| *Brevibacillus* brevis B011 | CP041767.1 | 99.74 |
| *Brevibacillus* brevis DZQ7 | CP030117.1 | 99.61 |
| *Brevibacillus* brevis HK544 | CP042161.1 | 100.00 |
| *Brevibacillus* brevis HNCS-1 | CP128411.1 | 99.61 |
| *Brevibacillus* brevis NBRC-100599 | AP008955.1 | 99.67 |
| *Brevibacillus* brevis NCTC2611-1 | LR134338.1 | 99.80 |
| *Brevibacillus* choshinensis HPD31-SP3 | CP069127.1 | 99.15 |
| *Brevibacillus* formosus NF2 | CP018145.1 | 99.74 |
| *Brevibacillus* parabrevis B3 | CP064090.1 | 99.22 |
| *Brevibacillus* parabrevis BCP-09 | CP118544.1 | 99.41 |
| *Brevibacillus* sp. DP1.3A | CP085876.1 | 99.74 |
| *Brevibacillus* sp. HD3.3A | CP085874.1 | 99.35 |

*Identity based on strains 16s BLAST against nr database (NCBI). All alignments had a sequence cover >99%.
